# Guiding functional reorganization of motor redundancy using a body-machine interface

**DOI:** 10.1186/s12984-020-00681-7

**Published:** 2020-05-11

**Authors:** Dalia De Santis, Ferdinando A. Mussa-Ivaldi

**Affiliations:** 1 Northwestern University and the Shirley Ryan AbilityLab, Chicago, IL USA; 2 Fondazione Istituto Italiano di Tecnologia, Genoa, Italy

**Keywords:** Body-machine interface, Motor redundancy, Forward model, Sensorimotor learning

## Abstract

**Background:**

Body-machine interfaces map movements onto commands to external devices. Redundant motion signals derived from inertial sensors are mapped onto lower-dimensional device commands. Then, the device users face two problems, a) the structural problem of understanding the operation of the interface and b) the performance problem of controlling the external device with high efficiency. We hypothesize that these problems, while being distinct are connected in that aligning the space of body movements with the space encoded by the interface, i.e. solving the structural problem, facilitates redundancy resolution towards increasing efficiency, i.e. solving the performance problem.

**Methods:**

Twenty unimpaired volunteers practiced controlling the movement of a computer cursor by moving their arms. Eight signals from four inertial sensors were mapped onto the two cursor’s coordinates on a screen. The mapping matrix was initialized by asking each user to perform free-form spontaneous upper-limb motions and deriving the two main principal components of the motion signals. Participants engaged in a reaching task for 18 min, followed by a tracking task. One group of 10 participants practiced with the same mapping throughout the experiment, while the other 10 with an adaptive mapping that was iteratively updated by recalculating the principal components based on ongoing movements.

**Results:**

Participants quickly reduced reaching time while also learning to distribute most movement variance over two dimensions. Participants with the fixed mapping distributed movement variance over a subspace that did not match the potent subspace defined by the interface map. In contrast, participant with the adaptive map reduced the difference between the two subspaces, resulting in a smaller amount of arm motions distributed over the null space of the interface map. This, in turn, enhanced movement efficiency without impairing generalization from reaching to tracking.

**Conclusions:**

Aligning the potent subspace encoded by the interface map to the user’s movement subspace guides redundancy resolution towards increasing movement efficiency, with implications for controlling assistive devices. In contrast, in the pursuit of rehabilitative goals, results would suggest that the interface must change to drive the statistics of user’s motions away from the established pattern and toward the engagement of movements to be recovered.

**Trial registration:**

ClinicalTrials.gov, NCT01608438, Registered 16 April 2012.

## Introduction

Injuries to the cervical spinal cord compromise function of the upper limb and result in severe deterioration of the quality of life. Functional recovery is not impossible, but is conditional to the amount of damage to the neural circuitry, which could worsen and consolidate with time [1]. Recent evidence [2–4] suggests that some of these changes might not be irreversible and that CNS remodeling is dependent upon the engagement of muscle groups in skilled activities and skill learning as opposed to passive repetition of movements or simple strength training [5–7].

The fact that plasticity is generally confined to the neuromuscular structures that were engaged in learning the sensorimotor skills, raises the question of how to involve in skilled activities movements or muscles for which residual voluntary control is severely limited by weakness or paralysis. A particular category of non-invasive interfaces that make use of wearable sensors, called body-machine interfaces (BoMI’s), was designed with the purpose of identifying movements that are still available to the paralyzed user and remapping them into a control input to a wide range of assistive devices [8, 9]. The general function implemented by a BoMI is of extracting the essential information encoded by multiple body signals so as to control a lower dimensional system of commands for operating an external device. This is a form of dimensionality reduction, where from a high dimensional signal space, a latent subspace of lower dimension is extracted by statistical methods such as principal component analysis.

Recently, BoMI’s have been proposed as instruments for functional retraining [10–12]. In the rehabilitative context, the BoMI can be configured to represent specific patterns of coordination that would be desirable for the user to practice/learn, that take the form of a forward map from body motion to effector positions.

The advantage of the BoMI systems is that the mapping can be tailored to the (dis)abilities of the user. The disadvantage, is that the burden of learning a suitable set of body postures or coordinated movements that actually can be used for controlling the system is left to the user. While the learning challenge is likely to be one of the key ingredients to the formation of a new motor repertoire, little is known about how these representations are formed and which learning principles govern them.

When the link between control inputs and sensory consequences is unknown, forward maps that allow predicting the effector position (the sensory target) corresponding to a specific body posture (the control input) are first learned through a process of exploration and then refined during a skill-acquisition phase [13, 14]. In situations when different patterns of control inputs yield to the same sensory feedback, the problem of identifying the body posture that corresponds to the desired effector position, that is constructing an inverse map [15], admits no unique solution. Hence, in the specific case of the BoMI, we expect users might adopt alternative coordination strategies to the ones encoded by the BoMI map. In addition, previous work has shown that the specific task requirements as well as rewards are important factors that contribute to guide strategy selection [16–18].

Given the complexity of the factors affecting learning novel sensorimotor maps and their interactions, we believe that a better understanding of how the brain resolves motor redundancy can provide powerful insights on how to design novel strategies for assistance and rehabilitation after spinal cord injury as well as other conditions affecting the motor systems.

In this work we are primarily interested in understanding the relationship between skilled performance and learning of a forward map when using a BoMI.

We first aim at identifying the possible mechanisms that the brain adopts when learning a novel sensorimotor transformation. In particular, we focus on two aspects, namely i) which criterion does the brain follow in developing novel coordination strategies and ii) how do task requirements influence the sensorimotor strategy adopted by the user.

The sensorimotor transformation was implemented here using a BoMI that mapped movement of the two arms of the user into movement of a 2D cursor. We evaluated learning by training unexperienced participants in controlling the cursor during a point to point reaching task. In order to study the effect of task requirements on movement strategies, we analyzed user motion when performing the trained reaching task and an untrained tracking task with the cursor.

Previous work [19–21] suggests that learning to control a 2D cursor with the BoMI results in both performance optimization in the trained task (faster reaching movements) and reduction of movement variance in dimensions that are not relevant to the task*.* In particular, the tendency of users to reorganize their movement along 2 dimensions or synergies has been taken as evidence of learning a forward map [20, 22]. *Here we take one further step and we hypothesize that performance improvement in the task are dependent on the identification of a suitable movement subspace and that greater improvements in performance are associated with learning of a more accurate forward model of the environment (HP1)*. *We further hypothesize that the forward map is specific to the learned task (HP2).*

If the first hypothesis is true, facilitating the development of a forward map can potentially result in a successful design strategy for increasing control efficiency, as measured by greater improvement in performance. Following this idea, we devised a method to facilitate the formation of a more accurate forward model by altering the BoMI map as learning proceeds. In particular, we propose to take advantage of the tendency of the CNS to reshape movement covariance during learning [23–25] to drive an online adaptation of the interface, a method originally introduced in [21]. Furthermore, if the second hypothesis is true, adapting the map would facilitate transfer of skills across tasks.

Finally, we propose that adaptation of the interface can also foster adaptation of the user and thus provide a powerful instrument for retraining specific motor patterns.

Some of the results presented here relative to 9 participants were previously published in a two-page abstract form as proceedings of the ICNR2018 conference [26].

## Materials and methods

### Experimental setup

**Fig. 1:**
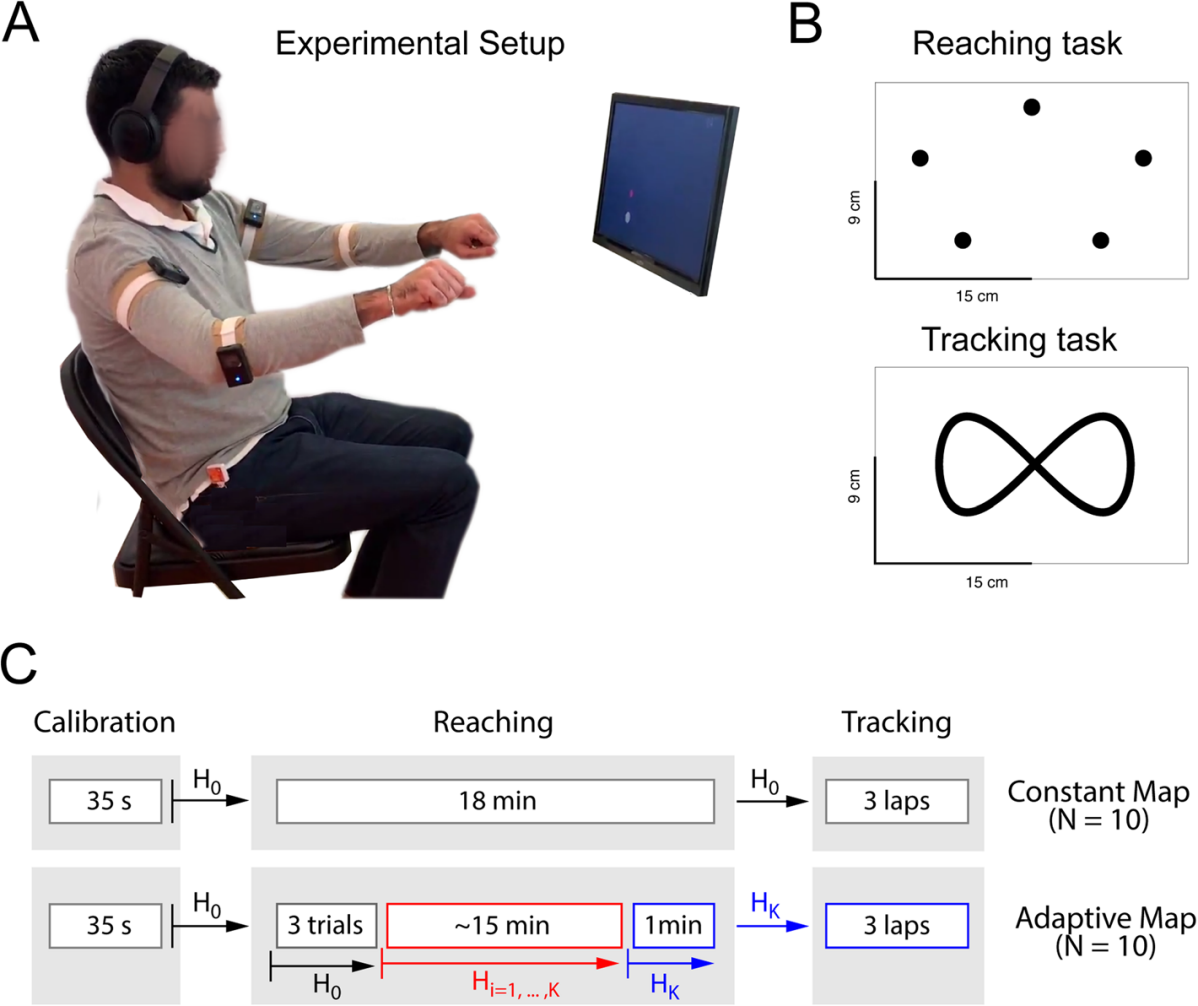
Experimental Setup and Protocol. Panel **a** – participants set in a chair with the sensors strapped around the arms and forearms as depicted, two per side, and were free to move their arms in a comfortable range. A screen positioned in front of the subjects displayed the cursor position, the current target position and a score in the top right corner. Panel **b** – target locations for the reaching task (top panel), and trajectory followed by the moving target during tracking (bottom panel). Panel **c** – summary of the experimental protocol for the Constant map group (top) and Adaptive map group (bottom). H_0_ stands for the BoMI map obtained after calibration, which is used by the Constant group throughout the session. H_i_ is the map updated iteratively during reaching in the Adaptive map group, and H_K_ is the final map after adaptation

Four Inertial Measurement Units (IMU) sensors (Three-Space Sensor™, Yost Labs Inc.) were strapped to the arms and forearms of the participant via Velcro® bands as in Fig. 1, panel a. Sensors were placed bilaterally, one proximally to the biceps, the other below the elbow on the ulnar bone. Pitch and Roll angles of each sensor were recorded using a custom written C++ code in Simulink®, yielding a total of 8 sensor inputs at a frequency of 50 Hz. The BoMI consisted of the *m*x*q* linear map *H* : *W*^*m*^ → *V*^*n*^ from the 8D vector space of arm motions, *q* ∈ *W*^*m*^, into the 2D vector space of cursor position on a computer screen, *p* ∈ *V*^*n*^, as defined by Eq. (1):

1 $$ \boldsymbol{p}=\mathrm{H}\left(\boldsymbol{s}-E\left[\boldsymbol{s}\right]\right) $$

The BoMI map *H*_*0*_ was defined via a simple calibration procedure. Each participant was instructed to move their arms spontaneously without task specifications in a comfortable range for 35 s. Subsequently, the dimensionality of the calibration dataset was reduced via Principal Component Analysis by extracting the first two components that expressed the greatest variance. H_0_ was thus defined to be the composition of the first two eigenvectors ***u***_**1**_***,u***_**2**_ of the calibration covariance matrix. It is important to note here that H_0_ : *W*_0_^*m*^ → *V*_0_^*n*^, where the vector space *W*_0_^*m*^ is the particular subspace of possible arm motions that have been observed during calibration.

This transformation allowed to map the first and second eigenvector onto width (x-axis) and height (y-axis) coordinates on the screen respectively. The cursor displacement was then rescaled by the eigenvalues *λ*_1_, *λ*_2_ and the width *w* and height *h* of the desired workspace:

2 $$ {\boldsymbol{p}}_{\mathbf{1}}={\boldsymbol{u}}_{\mathbf{1}}\boldsymbol{s}\frac{w}{\sqrt{\lambda_1}},\kern0.5em {\boldsymbol{p}}_{\mathbf{2}}={\boldsymbol{u}}_{\mathbf{2}}\boldsymbol{s}\frac{h}{\sqrt{\lambda_2}}; $$

The cursor was visualized on the screen as a red circle of 1 cm diameter whose position was controlled by different arm postures according to *H*_*0*_.

### Protocol

The study protocol was divided in three main phases. During the first phase, participants calibrated the interface using random arm motions, as described in the previous section. In the second phase, they practiced a reaching task to 5 target locations, shown in Fig. 1 – panel b, uniformly distributed on an ellipse with main axes 21.52 cm and 13.50 cm. The target location was displayed as a yellow circle of 1.5 cm diameter. The reaching task duration was set to 18 min. The practice was split into 4 blocks of respective duration 3′ – 5′ – 5′ – 5′. Participants were allowed to rest in between blocks as long as needed to reduce fatigue. During the third phase we evaluated participants’ ability to control the BoMI in an untrained novel task (tracking task). Participants were required to follow a target circle that moved along three complete laps of the infinity-shaped trajectory in Fig. 1 – panel b. The whole protocol lasted about 1 h and is summarized in Fig. 1 – panel c.

#### Reaching task

In the reaching task, participants were instructed to reach as many targets as possible in a given slot of time in order to maximize their score. For every target reached, the score would be incremented by 1 point. Furthermore, after the initial 12 trials, if the target was reached in less than 1.5 s, the score would be incremented by an extra point. Participants were given the score as a feedback at all times on the top-right edge of the screen. In order to reach a target, they had to hold the cursor within 1 cm of the target center for at least a second. One trial consisted of a complete reaching movement to a target.

Participants were also informed that on some trials the cursor position might not have been visible at the time of the target appearance. In this condition, which we called *blind trials*, participants were instructed to perform a quick motion that they thought would bring them to overlap with the target and hold the posture. After 2 s into the trial, the cursor would reappear to allow for correcting any offset. The absence of visual feedback in the first 2 s of movement allowed us to separate the effect of feedforward predictive control of the cursor movement from the corrective actions due to feedback mechanisms driven by visual error. Blind trials were interspersed pseudo-randomly in the target set.

#### Tracking task

During the tracking task, the target moved clockwise along an infinity-shaped trajectory at a rate of 60 s/lap. Participants were instructed to keep in the closest possible vicinity of the target, ideally overlapping with it at all times. Anytime the distance from the target fell below 5 cm, the target paused its motion.

In summary, participants engaged in three different tasks with the BoMI – a random movements task, a reaching task, and a tracking task. Random movements of the arms were used to calibrate the interface map H_0_. Participants only trained in the reaching task, and the tracking task was used to assess generalization of the reaching skills.

### Subjects and conditions

Twenty volunteers (age 19–45, 8F) with no history of neuromotor impairment participated in the study procedures. All participants gave their informed consent prior to the test. All procedures were carried out in accordance with the ethical standards of the IRB and with the Declaration of Helsinki and Northwestern University IRB approved all human involvement in the study (IRB #STU00057856).

Participants were split in two groups and assigned to one of the following study conditions, as in Fig. 1 – panel c:

i. *Constant map (group C, N = 10)*: participants were assigned a constant BoMI map H_0_, defined by an individual calibration procedure (see section 2.1). Therefore, each individual had a personalized map which did not change throughout the session.
ii. *Adaptive Map (group A, N = 10)*: the initial BoMI map H_0_ was defined by a calibration procedure as section 2.1. During the reaching task, an adaptive procedure (see section 2.4) continuously updated the body-cursor mapping in time as follows. The BoMI map was initialized with H_0_ and kept constant for 3 trials. From the 4th trial until 1′ to the end of the reaching task the map was iteratively updated. The final minute of training was then preformed keeping the map constant to the latest available map at the final time step K, H_K_. The same map H_K_ was also used during tracking.

### Strategy for adapting the map

Let us assume that learning to control a BoMI entails a progressive reshaping of movement coordination towards a set of “synergies” – or covariation patterns – that lie on a manifold that has reduced dimension. This assumption is consistent with previous studies that showed that learning novel redundant visuomotor mappings is accompanied by a progressive reduction in complexity of movement coordination [25, 27–29]. Moreover, we assume that the movement manifold is a linear subspace of all the possible movement vectors (each vector is one sensor orientation axis). In this sense, the BoMI implements one particular subspace of movement vectors, that we call H.

According to our first hypothesis (HP1), once BoMI users have identified a suitable movement subspace for controlling the interface, learning in terms of improving movement performance would proceed rapidly. Therefore, we devised a strategy for facilitating the identification of the BoMI manifold through its online reorientation to better match the natural movement manifold of the user as this evolves through exposure to particular tasks.

By construction, the BoMI subspace H represents the directions of maximum movement covariance over a set of calibration data. In practice, we expect movement covariance to have a different structure from calibration while the user learns to control the interface [26]. One possible way to compensate for the difference in distributions and to account for non-stationarity introduced by learning is recomputing the full covariance matrix using a batch of new data or a moving window on the data [30]. This operation, despite being beneficial to user’s performance [21], is however expensive in terms of memory consumption and computation time, which grows with the square of the batch size and the dimensionality of the data. Moreover, having to recompute the full covariance matrix through time with data available on the fly not only is expensive, but also unnecessary, given that we are only interested in the first few eigenvectors of movement covariance.

A more efficient and practical solution is given by iterative methods that approximate the eigenvectors of the covariance matrix without the need of having all the data readily available [31]. Weng et al. [32] proposed an iterative formulation that produces an average of a series of stochastic estimates of the eigenvectors of the underlying process covariance, without directly approximating the full covariance matrix. We summarized the method hereafter.

Let us consider a stochastic process with zero mean (without loss of generality) that generates a sequence of *N* independent identically distributed d-dimensional data **s**^(*k*)^***,****k* = 1, . . *N*, with unknown *dxd* sample covariance matrix Γ^(*N*)^. For a specific sample *k* in the sequence, Γ^(*k*)^ = *E*[ **s**^(*k*)^**s**^(*k*)*T*^], hence ${\Gamma}^{(N)}=\frac{1}{N+1}{\sum}_{k=1}^N{\Gamma}^{(k)}$. Knowing that the eigenvectors ***u*** and eigenvalues *λ* of the covariance matrix must satisfy the equality Γ**u*=****λ***u**, we can further write an iterative expression for **v**^(*N*)^ = *λ***u**^(*N*)^ given Γ^(*N*)^:

3 $$ {\mathbf{v}}^{(N)}=\frac{1}{N+1}{\sum}_{k=1}^N\ {\boldsymbol{s}}^{(k)}{\boldsymbol{s}}^{(k)T}{\mathbf{u}}^{(k)} $$

Where **u**^(*N*)^ = **v**^(*N*)^/‖**v**^(*N*)^‖, ‖**v**^(*N*)^‖ = *λ*. Finally, we can re-write equation (3) recursively for obtaining an update rule for the eigenvectors of Γ to apply iteratively for every new sample **s**^(*k*)^ as follows:

4 $$ {\mathbf{v}}^{(k)}=\frac{k-1}{k}{\mathbf{v}}^{\left(k-1\right)}+\frac{1}{k}\left(\ {\boldsymbol{s}}^{(k)}{\boldsymbol{s}}^{(k)T}\bullet \frac{{\mathbf{v}}^{\left(k-1\right)}}{\left\Vert {\mathbf{v}}^{\left(k-1\right)}\right\Vert}\right) $$

In order to recover orthogonality of the estimated eigenvectors, we can apply the Gram-Schmidt orthogonalization procedure on the vectors **v**^(*k*)^ obtained by equation (4). In the case of non-centered data, the sample mean can be estimated as ${\mathbf{m}}^{(k)}=\frac{k-1}{k}{\mathbf{m}}^{\left(k-1\right)}+\frac{1}{k}\ {\boldsymbol{s}}^{(k)}$**.**

From equation [7], we see that the estimate at the current time stamp k is composed of a memory element (the value of **v** at the previous step) and a novel element that depends on the mismatch between the sample covariance and the axes of the map and defines the direction of attraction of **v**. The effect of this computation is a progressive rotation of the original map towards the estimated directions of variance expressed by the user. The averaging component allows the formulation to be free of tuning parameters, as opposed to stochastic PCA approximations [31]. The drawback is that the estimate becomes progressively insensitive to new data as k increases, making the algorithm unsuitable for tracking non-stationary processes.

In order to cope with non-stationarity in the movement distribution, we have previously proposed a modified version of the iterative algorithm, that introduces the use of a fixed learning rate or amnesic parameter [32]. Considering a process with non-zero mean ***m*,** Equation (4) becomes as follows:

5 $$ {\mathbf{v}}^{(k)}=\left(1-\eta \right){\mathbf{v}}^{\left(k-1\right)}+\eta \left(\ {\boldsymbol{s}}^{(k)}-{\boldsymbol{m}}^{(k)}\right){\left({\boldsymbol{s}}^{(k)}-{\boldsymbol{m}}^{(k)}\right)}^{\boldsymbol{T}}\frac{{\mathbf{v}}^{\left(k-1\right)}}{\left\Vert {\mathbf{v}}^{\left(k-1\right)}\right\Vert } $$

6 $$ {\boldsymbol{m}}^{(k)}=\left(1-\eta \right){\boldsymbol{m}}^{\left(k-1\right)}+\eta\ {\boldsymbol{s}}^{(k)} $$

Equation (5) provides an updated estimate for the sample mean. The purpose of the amnesic parameter 0 < *η* < 1 in equation [5, 6] is to introduce an exponential downweighing effect over past samples so as to allow capturing new variation in the data. The estimate is subtracted to the data value at each iteration before computing the eigenvector update.

The amnesic parameter *η* can also be seen as the learning rate for map adaptation. The higher *η*, the faster new samples will steer the BoMI subspace towards recent experience. Schmitt et al. [33] suggested that in processes monitoring applications that make use of recursive PCA with exponential downweighing, the range of reasonable values for the amnesic parameter is commonly restricted to 0.9 < 1 − *η* < 0.9999, since values *η* > 0.01 might introduce instability in the model. On the other hand, values of *η* too close to 0 might lead to poor monitoring performance, which in our case translates in the algorithm having very low sensitivity to changes in covariance occurring at time scales faster than a few hours.

Convergence of non-adaptive formulation of the algorithm of equation (4) was provided in [34] under the assumption of Gaussian stationary processes. Even if convergence of equation (5) has not been mathematically proven yet, we have verified through simulations that the algorithm in equation (5) converges to the eigenvector of a series of template covariance matrixes in time for learning rates between 10^− 4^ and 10^− 2^. Learning rates in the range 0.001 to 0.003 yielded the best reconstruction in terms of estimated eigenvalues for a sample size of 500 to 3000 data points.

The problem of adopting learning rates that do not converge to zero with time has previously been addressed in other iterative formulations of the eigenvector problem since in practice the number will be nonzero due to numerical approximations and round-off errors [33]. In particular, [35, 36] provided proof of global convergence of the iterative PCA method of Oja and Karhunen [37], from which the algorithm of Weng et al. [32] was derived, in the case of constant learning rates. Other methods that employ recursive PCA showed that these algorithms are generally well behaved for a reasonable choice of the learning rate given the process dynamics. How to choose the optimal rate remains, however, an open problem.

In order to minimize sample-to-sample correlation when running the iterative PCA of equations (5, 6) online, we provided as input at each update step k the average of 5 samples of sensor data for which the sensor speed exceeded a threshold. This allowed suspending the update in conditions when no movement of the user’s arms was detected. After averaging, 500 and 3000 update steps correspond to roughly 1 and 5 min of continuous movement data when running at 50 Hz.

When selecting the learning rate, we assumed that the time scale of adaptation would differentially affect the development of a movement strategy. In particular, changing the map later into the training would not significantly affect the movement strategy, while a change early on would probably trigger a more significant co-adaptation. A previous study by Orsborne et al. [38, 39] suggested that the time scale of 1–2 min might be ideal when updating Kalman-filter based closed-loop decoders in brain machine interfaces in cases when the initial performance of the decoder is poor. Given the similarity of intents in our study, we followed a similar approach choosing *η* = 0.002 (time constant of approximately a minute).

### Outcome measures

During the reaching practice, we quantified performance over trials in terms of two metrics:

*Reaching Time* – the time from the first appearance of a new target to when the target has been reached successfully. This metrics was only computed when the cursor was visible for the whole duration of the trial. A reduction of Reaching Time indicates improvement in performance.

*Reaching Error* – the Euclidean distance between the target position and the cursor position after 2 s from target appearance. Since the distance across target location was not constant in our design, we normalized the Euclidean distance by the relative distance from the start target position and final target to be reached in a trial. Therefore, the measure has a lower bound of 0, in case the target was reached at or before 2 s. This metric was computed also for *blind trials*. In this case, the metrics quantifies the distance between the target and the cursor position at the time of cursor re-appearance.

We quantified performance in the tracking task in terms of average *Tracking Error*, which is computed as the Euclidean distance between the position of the moving target on the screen and the cursor position at a given instant of time.

We then computed a series of metrics to characterize the movement distribution (distribution of sensor values) in absolute terms (#1), relative to another distribution (#2) or relative to the BoMI manifold (#3):

*#1. Planarity Index* – the variance accounted for by the first 2 principal components of the sensor values over a certain period of time. Previous studies have shown that when learning a novel sensorimotor transformation, individuals tend to progressively distribute movement variance along dimensions that are task-relevant [25]. Given that the dimensionality of the task is only 2, we expected our participants to increase planarity in time. After computing the covariance matrix of the 8 sensor values **S** over a certain interval of time or trials, and denoting *λ*_*i*_ the eigenvalues of S, the Planarity index is computed as follows:

$$ \mathrm{Planarity}=\frac{\lambda_1+{\lambda}_2}{\sum_{i=1}^8{\lambda}_i} $$

*#2. Subspace Angle* – expresses the angle between two hyperplanes embedded in a higher dimensional space. If the two hyperplanes are parallel, the angle is zero. If they are orthogonal, the angle is 90 deg. Given two rectangular matrices 8 × 2 H_0_ and H_1_ whose columns are vectors of unit length, the Subspace Angle is computed as:

$$ \mathrm{Subspace}\ \mathrm{Angle}={\cos}^{-1}\left(\left|{H_0}^T{H}_1\right|\right)\bullet \frac{180}{\pi } $$

*#3. Variance accounted for by the BoMI (VAF)* – is the percentage of overall sensors variance accounted for by the 2D BoMI manifold and quantifies how much the movement distribution of the user is aligned with the 2D distribution defined by the BoMI. For the control group the BoMI manifold is a hyperplane defined by the calibration. For the Adaptive group, the BoMI hyperplane changes iteratively throughout the whole session as a function of the user’s movement distribution. The ideal value of 100% indicates that the movement variance is completely captured by the 2D BoMI manifold. VAF < 100% indicate that some components of variance project in the BoMI null space. Given the 8 × 8 covariance matrix of the sensor values **S** and the 2 × 2 covariance matrix of the corresponding cursor positions on the screen **C**, we computed the VAF as the ratio between the trace of C and the trace of S:

$$ \mathrm{VAF}=\frac{tr\left(\mathrm{C}\right)}{tr\left(\mathrm{S}\right)}\bullet 100 $$

### Data analysis and statistics

Sensors values and cursor positions were resampled in order to ensure a constant sample time and smoothed using a 3rd order Savitsky-Golay filter with 3 Hz cutting frequency. During reaching, trajectories in cursor and sensor spaces were segmented into trials excluding the holding phase on the target position. All analyses were carried out using MATLAB® R2017a.

Normality of the data was assessed using the Anderson-Darling test. When normality was met, we applied a t-test for comparing the performance of the two groups in the same block of trials or unit of time. Whenever we were interested in comparing the performance of one or both groups through time, we used a repeated measure analysis of variance (ANOVA) design with group as a between factor and repetitions as within factor. When sphericity was not met according to the Mauchly test, we reported the Greenhouse-Geisser correction for the *p*-values. If normality was not met, we applied a Mann Whitney U test for comparing the median of two populations. The repeated measures ANOVA was replaced by a Friedman’s ANOVA. The level of significance was set to 0.05. Post-hoc tests were carried out using the Tukey-Kramer procedure for multiple comparisons.

To compare the performance of participants in the beginning and the end of the reaching practice we computed the average Reaching Time and Reaching Error at 2 s during the first 20 and last 20 trials with complete vision of the cursor and Reaching Error at 2 s in the first and last 10 blind trials. To compare the evolution of movement distribution through time, we computed Planarity, Subspace Angle, and VAF metrics using the data from a 60 s time window either in the beginning or the end of the reaching practice. Whenever we were comparing performance metrics in trials with metrics qualifying movement distributions (i.e. Planarity), we used movement data within each trial that was included in the analysis.

In order to compute learning curves of Reaching Time and Planarity we followed two procedures.

#1: Computing learning curves across trials: here we were interested in obtaining a single model that could account for differences in learning dynamics within each experimental group. Hence, we used a non-linear mixed-effect model that accounts for inter-subject variability as a random factor affecting model parameters to fit a single exponential function to the Reaching Time and Planarity in first 90 trials with visual feedback (*nlmefit* function, Matlab 2017a Statistics and Machine Learning toolbox).

#2: Computing learning curves in time: in order to extract the dynamics of performance through time, we fitted an exponential model to the Reaching Time at each trial against the time elapsed from the beginning of the reaching practice to the end of the corresponding trial. Since we are here considering all the trials completed by a participant in the complete feedback condition, participants who completed greater amount of trials exhibited learning with different time-scales across trials of practice. For this reason, we fit two models to each individual’s data, a single and double exponential function using a non-linear least square regression (*fit* function, MATLAB 2017a) and we selected the model that yielded the higher adjusted R squared for each participant to extract the learning time constant(s).

## Results

### Performance in the trained task

**Fig. 2:**
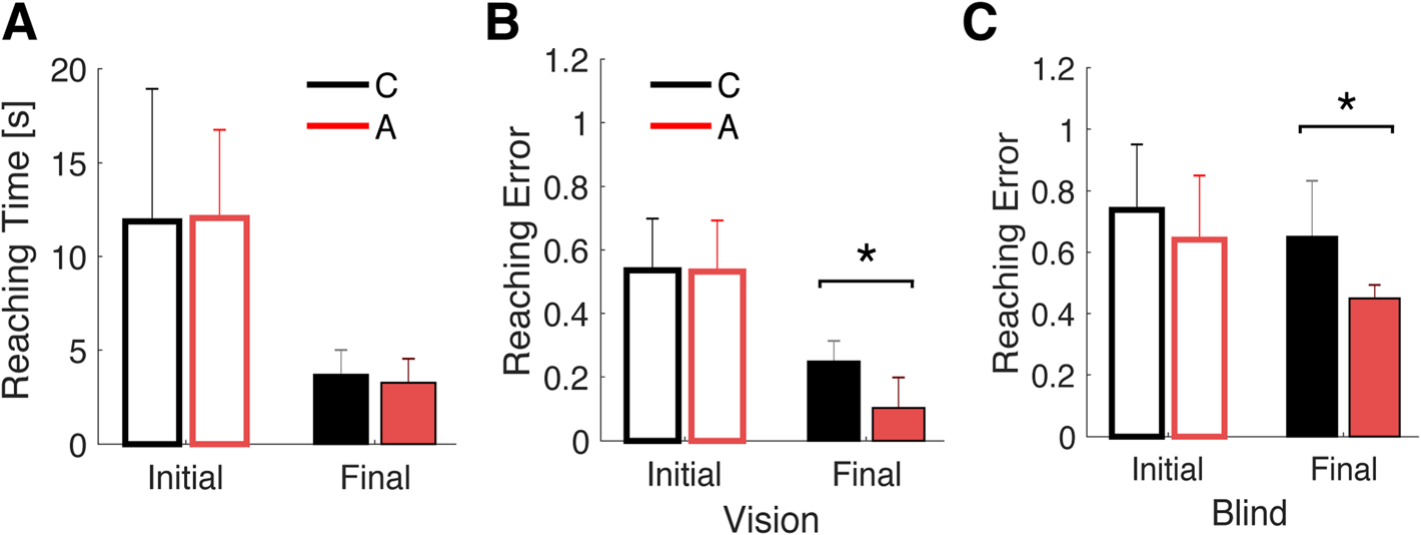
Summary of performance in the trained task. Panel **a** – average reaching time in the first and last 20 trials of reaching practice with full visual feedback of the cursor. Panel **b** – average Reaching Error during the first and last 20 trials with full visual feedback of the cursor. Panel **c** – Average Reaching Error in the first and last 10 trials in the blind condition. Black bars and red bars refer to Constant map and Adaptive map group respectively. The vertical error is the standard deviation, asterisks denote significant comparisons according to a t-test

In this section we first evaluate the performance of the two groups throughout the 18′ of practice while performing the reaching task. Figure 2 summarized the average performance over the first and last 20 trials for each participant for Reaching Time (panel a) and Reaching Error in the full visual feedback (panel b) condition and Reaching Error during the first and last 10 trials in the blind condition, when the cursor was invisible for the first 2 s of the trial (panel c).

Both Constant (C) and Adaptive (A) groups significantly improved their performance in terms of Reaching Time (C: *p* = 0.001, A: p < < 0.001) and no difference could be found between the two groups. However, the two groups were significantly different when considering Reaching Error at 2 s both during trials with full visual feedback and in trials when the visual feedback of the cursor was removed. Participants in the adaptive group were significantly more accurate at the end of the practice than the constant group (C vs A: Vision - *p* < 0.001; Blind - *p* = 0.003).

**Fig. 3:**
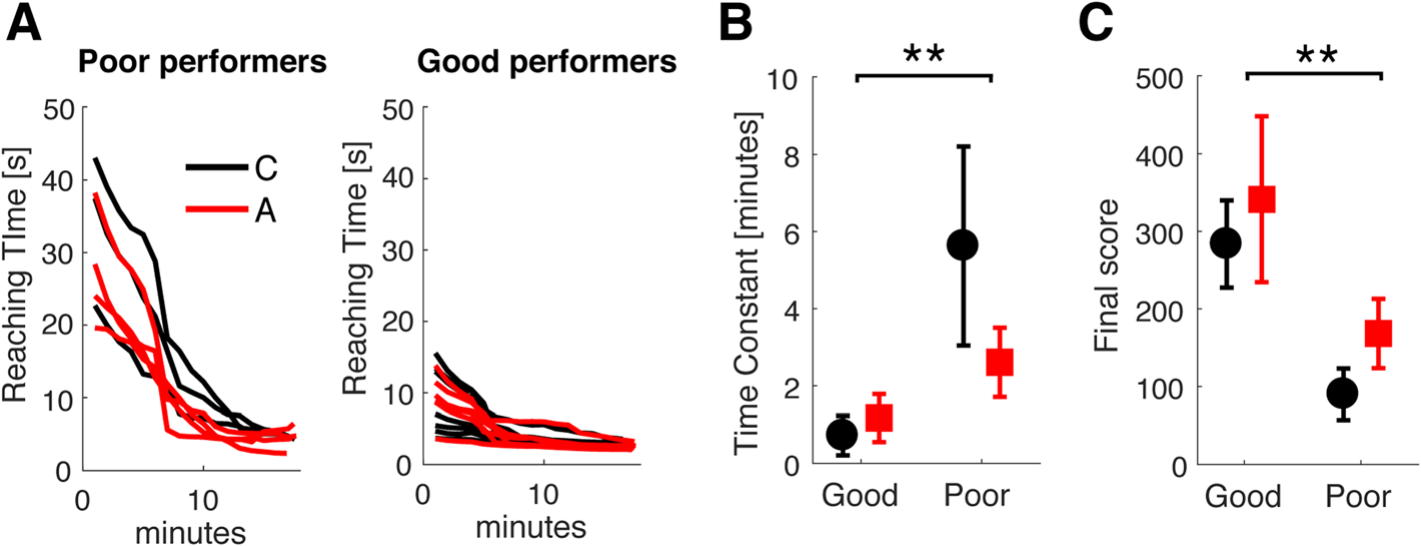
Participants grouping according to skill level. Panel **a** – Reaching Time for each trial through practice time for participants in the Constant (C - black lines) and Adaptive (A - red lines) map groups classified according to their initial performance in the reaching task. Panel **b** – average time constant estimated by fitting an exponential function to the Reaching Time over experiment time data in panel **a**. Panel **c** – average cumulative score across all blocks of reaching practice for good and poor performers, Adaptive map in red and Constant map in black. The vertical error is the standard deviation, asterisks denote significant comparisons according to a t-test ** for *p* < 0.001

Despite achieving similar group performance, the total number of trials completed by participants was greatly variable within each group (C: 193.2, min: 63, max: 260, A: 224, min: 134, max: 320]), as well as the individual score participants received as feedback during practice. Hence, we split participants in two groups of different skill level according to the amount of time it took for each of them to complete the first 5 trials. This value was extrapolated from a linear fit of the cumulative number of trials completed per unit time in the first 10 min of data. The population average 5-trial time was used as threshold for discriminating skilled vs less skilled participants. According to this criterion, 7 participants in the Constant map group and 6 in the Adaptive group were considered “good performers” (initial performance above average), while the remaining 3 and 4 participants respectively were considered “poor performers” (initial performance below average). Figure 3 depicts the Reaching Time according to the skill level for good and poor performers (panel a and b respectively). Each value is the average performance over a minute of practice. Finally, we computed the rate of improvement in performance over practice time by fitting an exponential function to the individual subject’s data.

As the panels b and c in Fig. 3 show, the two groups are well distinguishable both in terms of Reaching Time decay time constant (Good: C – 9 ± 4 s, A – 10.4 ± 3 s; Poor: C – 15 ± 1 s; A: 12 ± 6 s) and final score (Good: C – 284, A – 341; Poor: C – 90; A: 168). This difference is significant according to a factorial ANOVA over groups x skill level due to the participants’ skill, but no specific effect of the experimental condition was present (effect of skill – Time constant: F [1, 19] = 18.14, *p* = 0.0006; Score: F [1, 19] = 16.88, *p* = 0.0008, no effect of group – Time constant: F [1, 19] = 0.60, *p* = 0.449; Score: F [1, 19] = 0.57, *p* = 0.461, no skill x group interaction – Time constant: F [1, 19] = 1.49, *p* = 0.241, Score: F [1, 19] = 1.20, *p* = 0.289).

### Learning dynamics

**Fig. 4:**
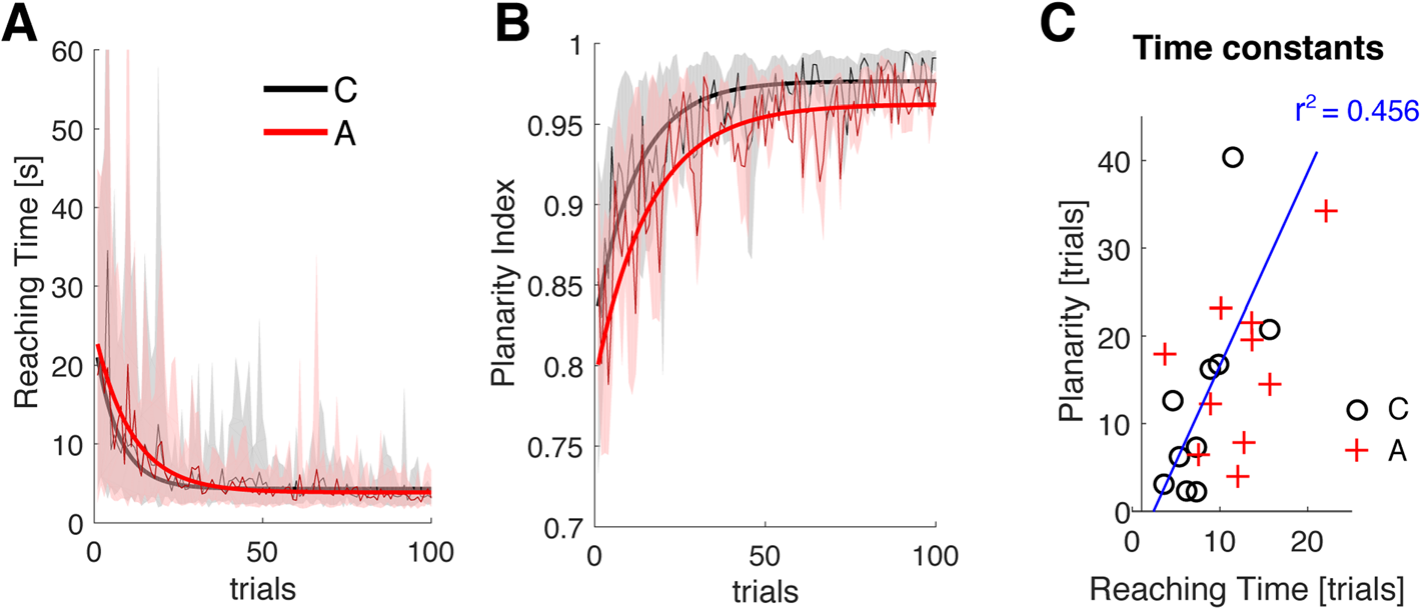
Learning dynamics. Panel **a** – average Reaching Time for the participants in the Constant group averaged in bins of 3 trials; Panel **b** – average Planarity Index of movement computed on bins of 3 trials. Shaded areas represent the 90% confidence interval around the mean. In black/red the result of fitting a single exponential model to the data of Constant/Adaptive map group using a non-linear mixed effect model; panel **c** – comparison of the time constants estimated by the model fitted on performance and on planarity for each individual in the two groups

Here we aim at comparing how learning in terms of performance proceeds relative to the development of a movement strategy consistent with the task requirements. Figure 4 shows the evolution of performance in terms of Reaching Time (panel a) and of the Planarity index (panel b) across trials for the Constant map group (in black) and the Adaptive group (in red). The solid lines represent the group model fitted by a non-linear mixed effect regression using a single exponential function to account for within-subject differences. Adaptation of the map did not impact the trial by trial evolution of performance or the planarity in the movement distribution (Average Model: Reaching time – C: 7 trials, A: 11 trials; Planarity – C: 12 trials, A: 16 trials). Notably, we found that improvement in Reaching Time was linearly related to an increase in movement planarity (Fig. 4 – panel c, *p* = 0.002, R^2^ = 45.6%). Indeed, participants whose movement variance was mostly distributed in two dimensions achieved a greater performance in the first 15 trials (*p* = 0.001). Planarity in the calibration dataset did not account for the difference in performance.

### Movement distributions in the trained task

**Fig. 5:**
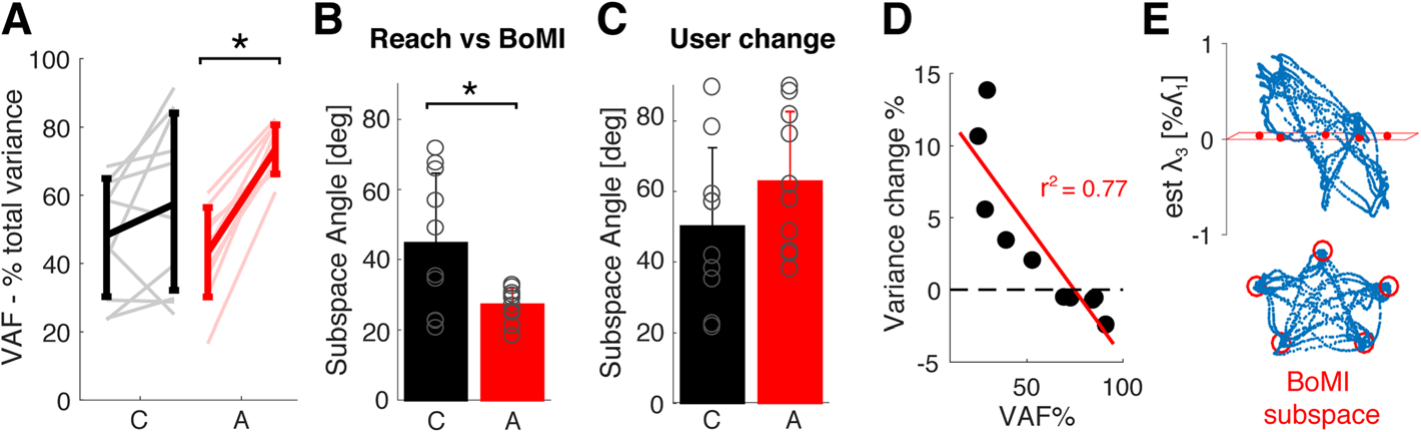
Movement distribution during reaching. Panel **a** - Variance Accounted for by the body-machine interface map in the first and last minute of reaching practice. Panel **b** – Subspace Angle between the subspace of reaching movements and BoMI subspace in the first and last minute of reaching. Constant map group is in black, adaptive map group in red. * represents significant differences after a paired t-test with *p* < 0.05. Panel **c** – Subspace angle between the two subspaces of reaching movements computed over the first and the last minute of reaching. Constant map group is in black, adaptive map group in red. Panel **d** – The X-axis represents the Variance Accounted for by the BoMI subspace in the last minute of reaching (as in panel A) for the participants of the Constant map group, while the Y-axis represents the percent change in movement variance during the last minute of reaching relative to the movement variance during calibration. The red line shows the linear model fit of the data. Panel **e** – Example of projection of the movement distribution during the last minute of reaching over the calibration subspace and over the subspace computed by taking the first two principal components of movement variance. The top figures show the projection in 3D using the 3rd principal component with variance *λ*_3_ as z-dimension, the bottom figures show the projection onto the first two principal components

We then quantified the effect of adapting the map in time on the movement distribution that the users adopt for solving the reaching task. The results are summarized in Fig. 5. Movement distributions of participants in the control group (in black), despite being mostly planar, have overall a small projection on the BoMI control manifold. Indeed, the percentage of variance accounted for by the 2D control manifold of the BoMI was only 58% on average in the last minute of training, and did not improve significantly with training (Fig. 5 – Panel a, VAF initial 60s – 48 ± 17%, *p* = 0.145). Similarly, the subspace angle between the 2D approximation of the movement distribution and the calibration manifold is generally high regardless of practice in the reaching task (Fig. 5 – Panel b, Subspace Angle – initial 60s – 60 ± 19 deg, final 60s: 46 ± 17 deg, *p* = 0.06). The difference in movement variance in the Constant map group relative to calibration is strictly correlated with the variance accounted for by the control map (R^2^ = 0.77, *p* < 0.001,) such that when VAF was low, participants compensated increasing movement variance. Figure 5 – panel d provides a visual example of the mismatch between the movement plane and the control plane obtained after calibration for a representative participant in the Constant map group.

Adapting the map, on the contrary, had a consistent and significant effect in increasing movement projection on the control plane and in reducing the distance between movement and control spaces (VAF – initial 60s: 43 ± 13%, final 60s: 74 ± 7%, p < < 0.001; Subspace Angle – initial 60s: 61 ± 14 deg, final 60s: 27 ± 5 deg, p < < 0.001). As a consequence of map adaptation, the two groups differ in terms of Subspace Angle, but no difference with VAF could be detected at the threshold of 0.05 due to the high inter-subject variability (VAF: *p* = 0.08, Subspace Angle: *p* = 0.015). Finally, we noticed that map adaptation still required participants to learn a preferred movement strategy, as suggested by the considerable change in the 2D approximation of user’s movement distribution in the first relative to the last minute of practice, regardless of the group (Fig. 5 – panel c, Subspace angle – C: 50 ± 23 deg, A: 63 ± 20 deg, *p* = 0.20).

### Generalization to a different task

**Fig. 6:**
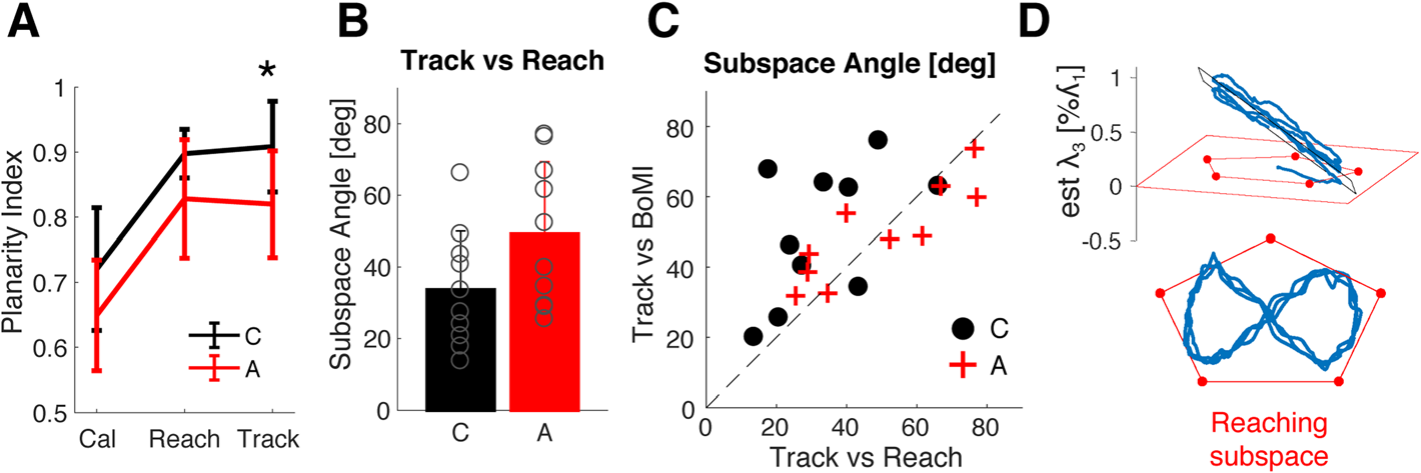
Effect of task on movement distribution. Panel **a** – Planarity Index computed across tasks: Cal – calibration, Reach = last minute of reaching, Track = tracking task. Constant map group is in black, Adaptive map group in red. * indicate significant differences with p < 0.05. Panel **b** – Subspace Angle between the subspace of tracking movements and the subspace spanned by reaching movements during the last minute of training, C = Constant map group, A = Adaptive Map group. Panel **c** – distance between tracking subspace and reaching subspace and the tracking subspace and the BoMI subspace in terms of Subspace Angle. The reaching subspace is computed during the last minute of training. Black dots are individual subjects in the Constant map group, Red crosses are individual subjects in the Adaptive map group. Panel **d** – example of tracking movement projected onto the subspace obtained from the last 60″ of reaching data in the space of the first 3 principal components (top view) and in the hyperplane of the first two

Performance in the untrained tracking task was not different between Constant and Adaptive map groups (Tracking Error – C: 1.4 ± 0.8 cm A: 1.4 ± 0.6 cm, *p* = 0.807). When comparing the subspace of arm movements during tracking to the BoMI subspace participants moved the cursor on during tracking, we saw no differences between the groups in terms of subspace angle (Subspace Angle – *Track* vs *BoMI* – C: 50 ± 19 deg, A: 50 ± 14 deg). However, participants in the Adaptive group tended to be significantly less planar than the Constant map group during tracking (*p* = 0.01), a difference not observed during calibration or reaching (Fig. 6, panel a).

Since we previously found that the movement subspace during reaching was generally different from the subspace of the BoMI map in the Constant map group (Fig. 5 – panel b), we compared the distribution of movements during the tracking task with the one participants developed in the end of the reaching practice. Figure 6 – panel d provides a visual example of a deviation from the reaching subspace during tracking in a representative participant of the Constant map group.

As Fig. 6 – panel b shows, the distance between tracking movements subspace and reaching movement subspace tended to be greater for the Adaptive map group than the Constant map group, even if not significantly (Subspace Angle – *Track* vs *Reach* – C: 34 ± 16 deg, A: 49 ± 20 deg, independent t-test *p* = 0.075).

Interestingly, we found that the movement subspace spanned during tracking by participants in the Control group was significantly closer to the subspace spanned in the end of the reaching practice than to the subspace of the BoMI (Subspace Angle – *Track* vs *BoMI* and *Track* vs *Reach* paired t-test: p = 0.01). Figure 6 – panel c highlights the comparison in terms of subspace angle between the subspace of tracking movements and i) the subspace of reaching movements (x-axis) or ii) the BoMI subspace (y-axis), for each participant in the Constant map (black circles) and Adaptive map (red cross) groups. Two observations can be made looking at Fig. 6 – panel c. The first is that the data of 8/10 participants in the Constant map group lies above the equality line, indicating a bias towards moving along the reaching subspace preferentially during tracking. Secondly, the distance between tracking and reaching subspace correlates with the distance between tracking and the latest BoMI used by participants (*p* = 0.001), such that moving further away from the BoMI map during tracking also meant deviating from the movements adopted during reaching.

## Discussion

The goal of this work was to design and test a method to adapt a human machine interface to its user’s evolving operational skill. The method is based on the relationship between the acquisition of a novel skill and learning of an internal model of action in the context of a redundant sensorimotor map. In the specific context of a body-machine interface [40] participants were asked to control the 2D position of a cursor on a screen by selecting appropriate arm postures in an 8D space. The overabundance of body dimensions allowed participants to choose amongst virtually infinite possible strategies that would be successful achieving the desired cursor positions. In mathematical terms, the BoMI mapping from body signals to cursor positions admits infinite inverse mappings, each inverse mapping corresponding to a representation of the task domain – i.e. the 2D space of cursor positions – in terms of 8D body signals. Among these representations one of importance is the “potent subspace” orthogonal at each point to the null-space of the BoMI mapping, that is to the space of body motions that do not cause any change in cursor position. If the movement distribution lies on the potent subspace of the BoMI map, no amount of motion is “wasted” along the orthogonal null space. Therefore, we predicted that movement distributions that lie closer to the BoMI’s potent subspace would lead to greater control ability. Accordingly, we compared the performance of participants practicing in either of two conditions. In the first, the BoMI subspace was fixed and the choice of an appropriate body map was left to the user (Constant map group). In the second condition the BoMI potent subspace would iteratively adapt to maximize the amount of the user’s movement projecting onto it (Adaptive map group) effectively reducing the distance between the user’s movement subspace and the BoMI potent subspace.

### Main take home message - factors affecting the ability to control a BoMI

Our results show that participants in the Adaptive group could control the cursor more accurately both with and without visual feedback of the cursor position. This result is consistent with the findings of our preliminary study [21], suggesting that map adaptation leads to forming a more accurate internal model and allows users to rely less on feedback mechanisms for achieving equal control ability.

Contrary to our expectations (HP1), we found that poor control ability in the Constant map group was not necessarily explained by a greater distance between the movement subspace and the potent subspace of the BoMI. Rather, the ability to distribute movement covariance along two dimensions, especially during the initial trials, predicted greater skill in point-to-point reaching with the cursor. In addition, even if participants learned to distribute movement variance on a plane within 10–20 trials, in contrast to earlier observations [25, 41] this plane did not necessarily converge to the BoMI potent subspace with further practice.

One possible interpretation of this result is that the variability in the movement subspace during reaching, as well as variability in skill level of participants, might result from differences in patterns of initial exploration. From a Bayesian point of view of learning, the prior distribution for movement selection is largely expressed by motor variability during the initial phases of learning [14, 42] and individual preferences in movement strategy [43]. For instance, in studying how people learn to control a planar manipulator with 3 joints using hand movements, Rohde et al. [44] found that individuals found solutions that were spatially biased towards the posture of the manipulator, hence their hand, practice was starting from. Moreover, they found that motor exploration of fingers’ degrees of freedom was tendentially structured rather than simply random, which is consistent with the hypothesis that motor exploration tends to occur along patterns that have been established by previous experience [20, 22].

According to this hypothesis, generalization of a skill to a different task will be facilitated when the novel task shares the same “structure” as the previous one. With a BoMI, we assume that the structure of the control task is dictated by the BoMI map, which in turn is associated with a linear 2D potent space embedded within a 8D space. Following this assumption, we can conclude that participants learned successfully one feature of the BoMI structure – its planarity, but they did not necessarily identify the potent subspace as preferred movement subspace. Rather, their internal model appeared to be calibrated on a “compatible” movement subspace, which also had a non-zero projection along the null-space of the BoMI. Indeed, when testing the generalization of reaching skills to the tracking task, we noticed that participants who trained with a constant map tended to distribute movement variance preferentially along this compatible subspace of reaching movements rather than along the BoMI’s potent space. Adaptation of the BoMI eliminated this bias, suggesting that map adaptation had the effect of reconciling the potent space of the BoMI with each user’s internal model. Despite this preferential bias towards the previously learnt movement distribution we found that the space of movement during tracking was in general not aligned with the space of movement during reaching, confirming our hypothesis that the forward map is task-dependent (HP2).

From our experiment we conclude that learning to perform a reaching task in a novel redundant environment was characterized by rapid improvements in performance in parallel with a fast increase in movement planarity from trial to trial. Whether these two processes that are active on a similar time-scale – one aiming at optimizing task performance, the other progressively reshaping movement distribution – interact with each other or are the separate products of a unique neural dynamics is still debatable. In fact, having to concurrently perform a goal-directed task introduces constraints – most notably in the sequence of errors and visited states – and biases the way the structural problem is solved [17, 44, 45]. Since reinforcement signals are associated with the specific task goals, the solution identified by the user is likely to differ from task to task [18]. The available action space might be as well affected by body constraints, such as specific impairments or limitations to movement. Furthermore, there are also limitations to the capacity of the neural substrate to reorganize patterns of activities, a concept also known as neural constraint [46–48]. Finally, external factors that manipulate feedback variability, such as the injection of noise, also bias the selection of a strategy over another [45, 49]. All the aforementioned factors and possibly others contribute to defining how motor strategies are selected amongst the available repertoire and their relation to the solution manifold of a specific task.

### Co-adaptation for assistance

Aside from identifying some of the possible mechanisms responsible for skilled performance when using a BoMI, the results of our study have several implications for using this type of interfaces in populations with motor impairments, such as individuals with cervical spinal cord injury. High-level injuries to the spinal cord not only leave the individual dependent on assistive devices life-long, but also severely limit the residual body’s dexterity.

Even though the distance between the BoMI’s potent subspace and the actual movement subspace was not able to account for differences in skill level across participants in the reaching task, it had a considerable impact on the movement strategy of the group that trained with a constant BoMI map. We noticed that when a consistent percentage of movement variance projected onto the null space of the BoMI map, participants reacted increasing the overall extent of their movements relative to calibration. We interpreted this result as an attempt to compensate for the loss of movement efficiency due to a poor alignment between their movement space and the BoMI’s potent subspace. This compensatory strategy is only feasible when users are actually able to increase their movement range. When the space of available movements is severely limited by paralysis or weakness, increasing range of motion might not be possible. In these situations, adapting the map to capture the directions of maximum variance in the movement would greatly benefit acceptance of the interface and partially alleviate the cognitive burden on the user in the initial phase of learning. More in general, loss of motor redundancy and range of motion due to paralysis, together with lack of experience performing control tasks with the degrees of freedom that were spared by the injury are likely to significantly constrain the search space, thus reducing the number of feasible solutions users can adopt. In this scenario, the cost for a bad initial guess could be significantly higher and lead to premature interface abandonment due to frustration.

Even if our study population had no motor impairment, we believe the results of this study would generalize well to a clinical population of individuals with spinal cord injury. SCI does not alter the capacity to adapt or learn a novel motor map [9], as the supraspinal neural architecture for motor learning is largely unaffected [50, 51]. On the other hand, BoMI’s are designed to account and compensate for individual’s residual mobility so as to overcome the influence of movement limitations on learning. Adaptation of the BoMI for the purpose of assistance, should concentrate on minimizing the differences between the user’s behavior and the interface.

### Co-adaptation for therapy

Previous studies reported that training upper body movements with a BoMI increased muscle strength and range of motion in individuals with complete injuries [9], improved symmetry and altered the pattern of muscle activity [11]. In addition, some preliminary studies suggested that the interface can be reprogrammed to progressively encourage participation in skilled activities of more distal parts of the body [10, 52]. These studies have shown that the mechanisms of motor learning can be exploited to compensate for changes in movement production following SCI by reassigning control authority over movements that are most deficient. A broad body of literature supports the idea that sustained engagement of sensorimotor systems in functional activities after SCI strongly facilitates plasticity in the nervous system, opening avenues for recovery [53–56].

Hence, we propose that interface adaptation could serve a dual purpose. Not only could the interface be programmed to better represent the user’s movement strategy in a specific task, but it could also be used as a tool to progressively enforce new patterns of mobility on the user. The results of our study point to the viability of this approach, as participants exhibited reactive behaviors in response to map adaptation. The difficulty in reducing movement planarity with practice (Fig. 6 – panel a) and the considerable change in movement strategy throughout the reaching task (Fig. 5 – panel c) suggest that users are not only sensitive to changes in the map but also active negotiators of a common control strategy. A previous work introduced the idea that rehabilitative goals can be encoded in the space of the BoMI [52]. They also showed how abrupt modifications of the BoMI map were effective in restoring movement symmetry in two individuals with complete cervical SCI. By following the same approach, we propose that map adaptation could lead incremental evolution of the interface towards encoded goals in the control space, driving user adaptation. The objective is to steer the control strategy away from undesired movement patterns toward practicing desired ones with the goal of functional retraining.

Other studies explored different strategies to encourage individuals to diverge from preferred movement strategies. One viable way to force participants to diverge from a preferred movement strategy is to make the visual feedback dependent on the distance from a selected strategy [16]. For instance, some studies manipulated visual feedback by associating greater noise to movement patterns that were undesirable [45, 49]. More in general, it has been shown that individuals quickly respond to perturbations in the map adapting their movement strategy [27] – or neural activation [51] – to recover high level of task performance. Previous work suggests that small gradual changes to the environment drive a progressive recalibration of internal models and the sensory inputs, leading to broader generalization than perturbations applied abruptly [57]. Together, these observations support the idea of applying adaptive interfaces for therapeutic purposes after SCI. Further studies will be investigating how to promote user adaptation by letting the map evolve towards new patterns of movement covariation.

### Co-adaptive interfaces and learning rate

Map co-adaptation in a BoMI closely parallels the problem of co-adaptation of a neural decoder in brain-machine interfaces, where the goal is to increase control ability by compensating for the mismatch in between open-loop (after off-line calibration) and closed-loop (during use) performance of the decoder in predicting the kinematic of a cursor [58]. This mismatch also engages neural adaptation in an attempt to compensate for poor initial performance of the decoder [47, 59] and to learn the imposed neural-to-kinematic mapping [50, 60].

Most of the approaches for decoder adaptation in brain machine interfaces make use of methods for adapting the parameters of Kalman filter equations that govern the neural to kinematics mapping [39, 61, 62]. These studies have shown that closed-loop decoder adaptation leads to increased performance during a reaching task. In this category of decoders, however, online cursor control requires the concurrent knowledge of the intended kinematic trajectory the user is trying to pursue. Hence, decoder adaptation might not be feasible when knowledge of the user’s goal is less clearly defined and reasonable estimates of user’s intention cannot be formulated. Other paradigms of co-adaptation make use of algorithms that aim at minimizing task-related error measures as in 2D cursor control by a myoelectric interface [63] and by remapping hand kinematics [64].

On the contrary, here we leave the problem of individuating a goal and generating a goal-directed movement to the user and we propose an unsupervised map adaptation which makes no assumptions of the specific requirements of the task the user is trying to perform and does not aim at compensating for errors in movement planning. It is important to stress the fact that map adaptation, as proposed here, still requires the user to actively learn the underlying structure of the BoMI and to adapt the internal model parameters to achieve high level of performance across different tasks. Hence, we believe that map adaptation only accommodates for individual strategies (or individual inverse models), allowing the user to still be actively engaged in the process of skill learning, a fundamental ingredient for promoting CNS plasticity [6, 11, 65].

### Shortcomings and limitations

We also need to highlight some shortcomings of the present work.

Our results demonstrated that map adaptation might not always be necessary. We argue that knowledge of the factors that are expression of learning in the context of redundant sensorimotor mappings can help identifying participants that might benefit from adapting the map the most and can inform the design of algorithms for modulating the rate of adaptation in time. For instance, participants whose movement variance projection onto the BoMI map was smaller might have benefited by higher learning rates initially to speed up alignment of the subspaces. A reduction in the learning rate later on in the practice might have been more beneficial for improving consolidation of the learnt strategy and promoting further increase in movement planarity.

A few other studies investigated how modulation of the learning rate and the choice of the time scale of adaptation influences performance of the decoder as well as the user in brain-machine interfaces. With the purpose of facilitating interface control, the learning rate should be chosen to balance fast improvements in performance and smoothness of interface changes. Dangi et al. [58] proposed a method to exponentially decrease the learning rate with time to ensure smoother convergence. Despite being a reasonable solution, this approach shifts the problem of tuning the learning rate to tuning the learning rate’s decay constant from case-to case. In another study, the learning rate is modulated according to the history of errors [63], allowing for faster adaptation in the presence of greater errors at any point during interface use (in a reaching task). Hence, this method incorporates knowledge of the task to compute a metric of error. Further work will be directed towards identifying ways to modulate the learning rate to maintain the unsupervised structure of the update algorithm.

Our results suggest that the time scale of map changes was appropriate for not interfering negatively with learning. We have previously verified that the amount of change in the BoMI map reduces with learning, thus indicating convergence of the map to a solution that likely derives from a negotiation process with the user [21]. However, a constant rate of adaptation might have amplified steady-state variability in the map in instances when convergence to a common solution was achieved. Adapting the map at a constant learning rate might have increased variability in the visual feedback of the cursor and thus have negatively impacted performance. A recent work by Cardis et al. [66] seem to suggest that variability has a negative impact on motor learning, regardless of its influence on task performance. Injecting variability in the BoMI map later on in the practice, might have contributed to promoting further motor exploration, limiting the ability to consolidate the movement strategy. Indeed, participants who practiced with the adaptive map exhibited less planarity in the movement distribution than their counterpart in the end of practice. Variability might have therefore slowed down the process of internalization of the map perhaps limiting retention of the skill across days, one hypothesis that we have not tested here. In our experiment, participants in the Adaptive group were nevertheless generally more accurate than the Constant map group, suggesting that the negative impact of variability on a shorter time scale, if present, was limited.

Part of the inter-subject variability observed in our experiment might depend on the choice of a customized interface calibration to preserve variability that naturally emerges across the users. In order to mitigate the effect of different initial conditions on our outcomes, we could have provided a fixed initial map for all participants, as we actually did in a previous preliminary study [21]. In that case we would have however incurred in a possible bias dictated by our particular choice of the map.

In addition, in order for participants in the adaptive group to experience some trials without map variation, we chose to freeze the map during the last minute of practice. It is possible that the choice of such short consolidation time impacted participants’ performance in the generalization task. Previous studies in a brain-machine interface suggest that prolonged training with a fixed decoder allows forming stable neural map of the decoder, which allows long-term retention and robust control [59].

Finally, we chose to initiate the algorithm’s update only after trial 3, to allow participants to explore the movement space in a static condition. We indeed hypothesized that most of the exploration would have occurred after the very first reaching trials. However, it is possible that our results are in part dependent on this choice. Further investigation is needed to understand the impact of initiating map adaptation earlier or later relative to the time-scale of learning.

## Conclusions

We studied how unimpaired subjects learn to solve the problem of redundancy in the solution space while learning a novel sensorimotor mapping encoded by a 2D BoMI. We identified two sub-problems, namely how individuals identify a suitable subspace of movements that can be used for control and how they enhance task-related performance. We found that subjects successfully learned to improve performance and also learned the task structure. We suggest that these two problems are solved in parallel and we proposed a method to improve accuracy of the newly formed forward model. We showed that aligning the subspace encoded by the BoMI to the movement subspace that emerges through learning allows guiding redundancy resolution towards increasing movement efficiency. We propose that a similar approach can be used as a strategy to first to encode preferred movement coordination patterns after SCI when controlling an assistive interface and later to guide the existing patterns toward new desirable ones by progressively shifting movement covariance.

## Data Availability

The datasets used and/or analyzed during the current study are available from the corresponding author on reasonable request.

## Abbreviations

BoMI: Body-machine Interface
CNS: central nervous system
PCA: principal component analysis.
IMU: inertial measurement unit
PI: planarity index
VAF: Variance accounted for or percentage of variance explained by a certain model or lower dimensional representation of a data distribution
